# Refining Risk Stratification in Light-Chain Cardiac Amyloidosis: A Single-Center Ambispective Cohort Study

**DOI:** 10.3390/diagnostics16193111

**Published:** 2026-09-24

**Authors:** Emre Ozerdem, Menekse Gerede Uludag, Seyhmus Atan, Halil Gulyigit, Müge Akbulut, Sibel Turhan, Irem Dincer, Güldane Cengiz Seval, Meral Beksac

**Affiliations:** 1 Department of Cardiology, Yıldırım Beyazıt University Yenimahalle Training and Research Hospital, 06370 Ankara, Türkiye; 2 Department of Cardiology, Ankara University Faculty of Medicine, 06230 Ankara, Türkiye; 3 Department of Cardiology, Kırıkkale High Specialty Hospital, 06230 Kırıkkale, Türkiye; 4 Department of Cardiology, University of Health Sciences Türkiye, İzmir Tepecik Training and Research Hospital, 35020 Izmir, Türkiye; 5 Department of Hematology, Ankara University Faculty of Medicine, 06230 Ankara, Türkiye; 6 Ankara Liv Hospital, Istinye University, 06680 Ankara, Türkiye

**Keywords:** AL amyloidosis, cardiac amyloidosis, prognosis, systolic blood pressure, survival

## Abstract

**Background:** Recent advances in disease awareness and diagnostic strategies have led to earlier diagnosis and improved survival in patients with light-chain cardiac amyloidosis (AL-CA). However, currently used prognostic staging systems are based on older cohorts and rely mainly on cardiac biomarkers, without incorporating patient-related clinical parameters. **Aims:** We sought to evaluate contemporary survival outcomes in patients with AL cardiac amyloidosis and to identify clinically available predictors that may improve current risk stratification. **Methods:** This was a single-center ambispective cohort study including consecutive patients diagnosed with AL cardiac amyloidosis between 2014 and 2024. **Results:** Ninety-six consecutive patients with AL-CA diagnosed between 2014 and 2024 were included. The mean age was 59.6 ± 11.2 years, and 38 patients (39.6%) were women. During follow-up, 51 patients (53.1%) died. Multivariable Cox regression identified lower systolic blood pressure (SBP) (HR 1.30, 95% CI 1.10–1.54; *p* = 0.002), troponin levels >3× the upper reference limit (HR 2.67, 95% CI 1.29–5.55; *p* = 0.008), and advanced New York Heart Association (NYHA) functional class (HR 1.92, 95% CI 1.14–3.24; *p* = 0.014) as independent predictors of mortality. Adding SBP to the Mayo 2012 staging system was associated with a modest improvement in prognostic discrimination (C-index: 0.685 vs. 0.723; ΔC-index = 0.038) and a modest reduction in prediction error. **Conclusions:** Lower SBP, marked troponin elevation, and advanced NYHA functional class are independent predictors of mortality in AL-CA. SBP may provide modest incremental prognostic information beyond the Mayo 2012 staging system; however, its added predictive value requires validation in larger independent cohorts.

## 1. Introduction

Cardiac amyloidosis (CA) is a progressive cardiomyopathy characterized by the accumulation of misfolded endogenous proteins in the form of amyloid fibrils within the heart which leads to diastolic dysfunction in the early stages and systolic dysfunction in the advanced stages [1,2]. Conventionally regarded as a rare and difficult-to-treat disease, cardiac amyloidosis has undergone a paradigm shift in perception over the past few years due to significant advances in diagnostic and therapeutic strategies, thereby generating increasing interest within the scientific community [3].

More than 95% of cardiac amyloidosis cases diagnosed today arise from either AL or transthyretin-derived fibrils [2,4]. AL amyloidosis is a rare, progressive, systemic disease more frequently observed in men. In AL amyloidosis, the nonspecific nature of symptoms often leads to delays in diagnosis, which in turn result in cumulative organ damage, poorer prognosis, and early death [5]. Cardiac involvement, in particular, serves as a marker of poor prognosis, with approximately 40% of patients succumbing within the first year following diagnosis. This may contribute to an underestimation of the actual prevalence of AL-CA [6]. However, increasing awareness has led to a parallel increase in diagnosis rates.

Previous studies have investigated multiple parameters to predict prognosis in patients with CA. For both AL-CA and TTR-CA, troponin and N-terminal pro-B-type natriuretic peptide (NT-proBNP) are strong indicators of disease burden and prognosis [7,8]. Multistage staging systems, predominantly based on these biomarkers, have been developed [9,10,11].

Current prognostic staging systems for AL-CA were developed using historical cohorts before the era of effective therapies and rely exclusively on cardiac biomarkers, highlighting the need for contemporary refinement. This study aimed to investigate the clinical parameters that influence survival in patients diagnosed with AL-CA in our clinic.

## 2. Methods

This was a single-center ambispective cohort study including 96 consecutive patients diagnosed with AL cardiac amyloidosis between 2014 and January 2024. All eligible patients were retrospectively identified from institutional medical records. Following ethics committee approval on 28 March 2024, surviving patients were followed prospectively through routine clinical visits and telephone contacts until January 2026.

The last patient was diagnosed in January 2024, and follow-up was completed in January 2026. Vital status was ascertained through institutional hospital records and telephone follow-up. Patients who remained alive at the end of follow-up were censored at their last confirmed follow-up. The median follow-up duration, estimated using the reverse Kaplan–Meier method, was 48 months. Baseline clinical, laboratory, and echocardiographic variables were obtained at the time of AL-CA diagnosis, and the date of diagnosis was used as the uniform index date for all patients.

The Ankara University Faculty of Medicine Clinical Research Ethics Committee (Decision No: İ03-237-24) approved the study. For the retrospective component of the cohort, the requirement for informed consent was waived by the Ethics Committee. Written informed consent was obtained from patients who participated in prospective follow-up after ethics committee approval. The study was conducted in accordance with the ethical standards and principles of the Declaration of Helsinki.

The diagnosis of AL cardiac amyloidosis was established in accordance with the diagnostic algorithms outlined in the most recent consensus reports of the European Society of Cardiology (ESC) and the American College of Cardiology (ACC) [2,12]. Cardiac involvement was established by the presence of typical echocardiographic and/or cardiac magnetic resonance (CMR) findings together with histopathological evidence of amyloid deposition on extracardiac tissue biopsy, or by direct demonstration of cardiac amyloid deposition on endomyocardial biopsy. As part of the routine diagnostic work-up for the underlying plasma cell disorder, all patients underwent serum and urine immunofixation and serum free light-chain assessment. Bone marrow evaluation was routinely performed, with bone marrow biopsy performed in 75 patients (78.1%). Additional extracardiac tissue sampling included abdominal fat pad or renal biopsy, as clinically indicated. Endomyocardial biopsy was performed in 3 patients (3.1%), and CMR was performed in 41 patients (42.7%).

Disease severity was classified according to the Mayo 2012 staging system using three prognostic markers: NT-proBNP ≥ 1800 pg/mL, dFLC ≥ 18 mg/dL (180 mg/L), and the corresponding cardiac troponin threshold. For patients with hs-cTnT measurements, a threshold of ≥40 ng/L was used, corresponding to the conventional cTnT threshold of ≥0.025 μg/L, as previously proposed. For patients in whom only cTnI was available, a threshold of ≥0.1 ng/mL was used based on the assay-specific threshold reported in the established AL amyloidosis staging literature. One point was assigned for each marker meeting the specified threshold, with scores of 0, 1, 2, and 3 corresponding to Mayo stages 1, 2, 3, and 4, respectively. Because cTnI does not have a formally established equivalent threshold within the Mayo 2012 model, a sensitivity analysis was additionally performed in patients with available hs-cTnT measurements.

Separately, for the analysis of marked troponin elevation as a prognostic variable, troponin concentrations were standardized according to the assay-specific upper reference limit (URL) because different cardiac troponin assays were used during the study period. Marked troponin elevation was defined as >3× the assay-specific URL. The URLs were 14 ng/L for hs-cTnT and 0.06 ng/mL for cTnI, corresponding to >3× URL thresholds of >42 ng/L and >0.18 ng/mL, respectively.

NYHA functional class was determined at the initial clinical evaluation based on patients’ symptoms and functional capacity according to standard NYHA criteria. In the Cox regression analysis, NYHA functional class was entered as an ordinal variable, with the hazard ratio representing the effect of each one-class increase.

Echocardiographic evaluations were performed using a GE Vivid E9 system (GE Healthcare, Horten, Norway). Left ventricular mass was calculated using the Devereux formula [13]. Myocardial contraction fraction (MCF) was calculated as stroke volume divided by myocardial volume (MV), with MV calculated by dividing LV mass by the assumed myocardial density of 1.05 g/mL [14]. The LVEF/LVGLS ratio was calculated by dividing LVEF by the absolute value of LVGLS. The apical-to-basal strain ratio was calculated as the ratio of average apical longitudinal strain to average basal longitudinal strain, using absolute strain values.

### Statistical Analysis

All statistical analyses were performed using IBM SPSS Statistics version 25.0 (IBM Corporation, Armonk, NY, USA). A *p*-value of <0.05 was considered statistically significant. The distribution of continuous variables was assessed using the Kolmogorov–Smirnov test, while homogeneity of variances was evaluated using Levene’s test. Descriptive statistics were expressed as mean ± standard deviation, median (minimum–maximum), or median [25th–75th percentile] for continuous variables, and as frequency and percentage for categorical variables.

The primary endpoint of the study was overall survival (OS), defined as the time from the date of AL-CA diagnosis to death from any cause. Patients who remained alive were censored at the date of their last confirmed follow-up. Kaplan–Meier survival analysis was used to estimate OS across different subgroups based on Mayo staging and a combined classification incorporating Mayo stage and systolic blood pressure. Comparisons between groups were conducted using the Log-Rank test. Crude survival rates at 6 months, 1 year, 2 years, and 5 years, as well as median survival times with corresponding 95% confidence intervals, were calculated for each subgroup. To assess the incremental prognostic value of SBP beyond the Mayo 2012 staging system, Harrell’s concordance index (C-index) was calculated for the Mayo 2012 model alone and after the addition of SBP ≤100 mmHg. The change in C-index (ΔC-index) was calculated, and its 95% confidence interval was estimated using 3000 paired bootstrap resamples. In addition, the nested Cox models were compared using the likelihood-ratio test.

Univariate analyses were performed using Cox proportional hazards regression to identify factors potentially associated with OS. Variables with *p* < 0.10 in univariate analysis were included in a multivariate Cox regression model using the backward stepwise (likelihood-ratio) method. Variables deemed clinically irrelevant, or those with missing data or multicollinearity issues, were excluded from the final model. For each variable, hazard ratios (HRs), 95% confidence intervals, and Wald statistics were reported. Given the wide and right-skewed distribution of NT-proBNP values, an additional Cox regression analysis was performed using the established Mayo 2012 cut-off of ≥1800 pg/mL.

The SBP threshold of ≤100 mmHg was selected a priori based on previously reported prognostic thresholds in AL-CA populations [15,16], rather than being derived from the present dataset. SBP was also analyzed as a continuous variable to avoid loss of prognostic information associated with dichotomization. Missing data were handled using a complete-case approach according to the variables included in each analysis, and no imputation was performed. Missingness primarily reflected unavailable measurements in the medical records, particularly for selected echocardiographic parameters. The functional forms of continuous predictors were examined using restricted cubic spline models. No significant evidence of nonlinearity was identified for SBP, NT-proBNP, dFLC, uric acid, LVEF, or GLS; therefore, these variables were retained as continuous linear terms in the Cox regression analyses.

## 3. Results

The mean age of the patients with CA in our study was 59.6 ± 11.2 years. The demographic characteristics of patients are shown in Table 1.

Of the 96 patients, 79 (82.3%) had primary AL amyloidosis, whereas 17 (17.7%) had multiple myeloma-associated AL amyloidosis. hs-cTnT (reference range, 0–14 ng/L) and cTnI (reference range, 0–0.06 ng/mL) were measured in 70 and 26 patients, respectively. Among the 70 patients with hs-cTnT measurements, 48 (68.6%) had hs-cTnT levels >3× the assay-specific upper reference limit (URL), whereas 14 of the 26 patients (53.8%) assessed with cTnI had cTnI levels >3 × URL. In a sensitivity analysis restricted to patients with hs-cTnT measurements, hs-cTnT >3 × URL remained significantly associated with mortality (HR 6.83, 95% CI 2.07–22.56; *p* = 0.002).

The median follow-up duration, estimated using the reverse Kaplan–Meier method, was 48 months. During follow-up, 51 patients (53.1%) died. The distribution of NYHA functional class was as follows: 7 patients (7.3%) were classified as NYHA class I, 48 (50.0%) as class II, 30 (31.3%) as class III, and 11 (11.5%) as class IV.

The echocardiographic characteristics of the study population are summarized in Table 2.

During follow-up, 51 of 96 patients (53.1%) died. Kaplan–Meier-estimated overall survival probabilities at 6 months, 1 year, 2 years, and 5 years were 69.8%, 58.2%, 51.9%, and 42.8%, respectively (Figure 1). The Kaplan–Meier-estimated median overall survival was 36 months.

In a sensitivity analysis according to calendar period, 38 patients were diagnosed in 2014–2018 and 58 in 2019–2024, with 25 and 26 deaths, respectively. Diagnosis in 2019–2024 was not significantly associated with overall survival compared with 2014–2018 (HR 0.78, 95% CI 0.45–1.38; *p* = 0.396).

In the AL-CA group, univariable Cox proportional hazards regression analysis of all factors considered to potentially affect overall survival revealed that lower systolic blood pressure, higher NYHA class, troponin levels at diagnosis exceeding three times the upper reference limit, and increases in uric acid and dFLC levels were associated with a statistically significant increase in mortality rate (Table 3). NT-proBNP data were available in 92 patients (95.8%). Although NT-proBNP analyzed as a continuous variable was not significantly associated with overall survival, NT-proBNP ≥ 1800 pg/mL was significantly associated with increased mortality (HR 2.22, 95% CI 1.16–4.26; *p* = 0.017).

In addition, an increase in the LVEF/strain ratio, a decrease in LVGLS values, and the presence of low voltage on ECG were associated with a statistically significant increase in mortality rate (Table 4). However, when these parameters were included in the multivariable analysis, they lost their statistical significance.

Mortality rate increased statistically significantly with each stage increase in Mayo staging (*p* < 0.001). According to the Mayo staging system, there was a statistically significant difference in overall survival (*p* = 0.002), which was attributable to a poorer prognosis in patients with Mayo stage 4 compared to those with stages 1, 2, and 3 (*p* = 0.004, *p* = 0.005, and *p* = 0.040, respectively) (Figure 2). In the Mayo stage 4 group, the mean survival was 23.2 months while the median survival was 6 months.

Based on the results of the multivariable backward stepwise Cox proportional hazards regression analysis, the most significant predictors of overall survival were, in order of importance: lower systolic blood pressure, troponin levels exceeding three times the reference limit, and higher NYHA class. A 10 mmHg decrease in systolic blood pressure was associated with a significantly increased risk of mortality [HR = 1.30, 95% CI: 1.10–1.54, *p* = 0.002]. Similarly, troponin levels greater than three times the normal limit [HR = 2.67, 95% CI: 1.29–5.55, *p* = 0.008] and each incremental increase in NYHA class [HR = 1.92, 95% CI: 1.14–3.24, *p* = 0.014] were significantly associated with higher mortality risk (Table 5).

Then, SBP was incorporated into the Mayo 2012 staging system. Statistically significant differences in overall survival were observed across the resulting subgroups (*p* < 0.001). The main difference was the worse prognosis observed in patients classified as Mayo stage 3–4 with SBP ≤ 100 mmHg compared with the subgroups of Mayo stage 1–2 with SBP > 100 mmHg, Mayo stage 1–2 with SBP ≤ 100 mmHg, and Mayo stage 3–4 with SBP > 100 mmHg (*p* < 0.001, *p* = 0.030, and *p* = 0.011, respectively) (Figure 3). Harrell’s C-index increased from 0.685 for the Mayo 2012 staging system alone to 0.723 after the addition of SBP ≤ 100 mmHg (ΔC-index = 0.038; bootstrap 95% CI, 0.006–0.090), indicating a modest improvement in discrimination. Prediction error was also modestly reduced, with the integrated Brier score decreasing from 0.200 for the Mayo-only model to 0.188 for the Mayo-plus-SBP model over 5 years. At 1 year, the corresponding Brier scores were 0.215 and 0.204, respectively. One-year calibration-in-the-large values were 0.196 and 0.192 for the Mayo-only and Mayo-plus-SBP models, respectively. The likelihood-ratio test comparing the nested models was statistically significant (χ^2^ = 4.50, *p* = 0.034).

In a sensitivity analysis restricted to patients with available hs-cTnT measurements, complete Mayo 2012 staging data were available for 65 patients, among whom 27 deaths occurred. Mayo 2012 stage remained significantly associated with mortality (HR 2.32, 95% CI 1.43–3.77; *p* < 0.001). After addition of SBP ≤ 100 mmHg, both Mayo stage (HR 1.95, 95% CI 1.19–3.17; *p* = 0.008) and SBP ≤ 100 mmHg (HR 2.94, 95% CI 1.15–7.52; *p* = 0.024) remained associated with mortality. The C-index increased from 0.700 for Mayo stage alone to 0.747 after addition of SBP (ΔC-index = 0.047), and model fit improved (likelihood-ratio χ^2^ = 5.58, *p* = 0.018).

## 4. Discussion

Most prognostic data in AL-CA are derived from historical cohorts that predate current therapeutic advances. Treatment strategies for AL amyloidosis evolved during the study period, particularly with the increasing use of daratumumab-based regimens. However, detailed treatment data were unavailable; therefore, their potential impact on survival could not be evaluated.

In 2019, Barrett et al. evaluated 194 patients with systemic AL amyloidosis and reported markedly improved survival compared with earlier studies, with a mean survival of 118, 76, 64, and 27 months for Mayo stages 1–4, respectively. Survival improved more than threefold in patients with Mayo stage 2 and 3 disease compared with the 2012 Mayo cohort. Although median survival in stage IV remained limited to 6 months, a mean survival of 27 months suggests that modern therapies have substantially improved outcomes, even in advanced-stage disease. These findings underscore the need for contemporary studies to reassess survival and prognostic determinants in the current treatment era [17].

The mortality rate observed in our cohort between 2014 and 2024 was comparable to that reported by Barrett et al. The median life expectancy of our patient population was 36 months (95% CI: 6.76–65.2). In addition, the crude mortality rate was 53.1% and it was observed that approximately 40% of deaths occurred within the first year. Consistent with the findings reported by Barret et al., the median survival in the Mayo stage 4 group was 6 months, while the mean survival was 23.2 months. Both the data from Barret et al. and our results indicate that survival outcomes have improved even among patients with advanced CA.

Current prognostic staging systems for AL-CA, including the Mayo staging system, rely mainly on cardiac biomarkers and do not incorporate patient-related clinical parameters [18]. In cardiac amyloidosis, the deposition of amyloid fibrils in the myocardium affects the relaxation and contraction function of the myocardium, blood pressure levels decrease with a decrease in stroke volume, and intolerance to antihypertensive agents increases [19]. In a prospective study examining the effect of blood pressure levels on prognosis in AL-CA patients, lower SBP levels were associated with lower survival. In the study, statistically significant higher mortality was observed within 6 months in patients with BP below 100 mmHg and within 2 months in patients with BP below 90 mmHg [15]. In another European-centered MAYO study evaluating stage 3 AL-CA patients including 346 patients, SBP less than 100 mmHg was found to be an independent predictor of poor survival [16]. Similarly, systolic blood pressure was identified as an independent predictor of overall survival in our study. When SBP was incorporated into the Mayo 2012 staging system, low SBP (≤100 mmHg) was particularly associated with poorer survival among patients with advanced Mayo stages (3–4). The addition of SBP ≤ 100 mmHg increased Harrell’s C-index from 0.685 to 0.723 (ΔC-index = 0.038) and was associated with a modest reduction in prediction error. However, the absolute improvement in discrimination was limited. Therefore, these findings should be interpreted as preliminary evidence that SBP may provide incremental prognostic information beyond Mayo staging, particularly in patients with advanced-stage disease, rather than as proof of a clinically meaningful improvement in prediction. Validation in larger independent cohorts is warranted.

Elevated cardiac troponin levels have been consistently associated with poor prognosis in AL-CA. Dispenzieri et al. reported worse survival in patients with elevated troponin T and I levels. TnT has emerged as a better predictor compared to TnI in studies [7]. Different thresholds for cTnT, hs-cTnT, and cTnI have been presented in many different staging scores. For instance, the 2004 Mayo staging system specified a threshold of ≥50 ng/L for TnT, while the threshold for hs-cTnT was set at ≥40 ng/L in Mayo 2012. Different threshold values have been defined for TnI depending on the assay kit used [20]. Given that the use of different cut-off values across staging systems may introduce limitations in patient stratification, in this study we examined the impact of troponin levels at diagnosis without applying a universal threshold. Specifically, we evaluated whether troponin levels exceeding three times the assay-specific reference limit were associated with overall survival. Marked troponin elevation (>3× the assay-specific URL) was associated with worse overall survival in univariable analysis (HR 3.36, 95% CI 1.70–6.63; *p* < 0.001) and remained independently associated with mortality in the final multivariable Cox model (HR 2.67, 95% CI 1.29–5.55; *p* = 0.008).

NT-proBNP is a well-established prognostic biomarker in AL-CA and constitutes an important component of the Mayo 2012 staging system. In our cohort, NT-proBNP analyzed as a continuous variable was not significantly associated with overall survival. However, when evaluated using the established Mayo 2012 cut-off, NT-proBNP ≥ 1800 pg/mL was significantly associated with increased mortality. This finding supports the prognostic relevance of NT-proBNP in our cohort and suggests that its prognostic association may be better captured using the established clinically validated threshold.

Elevated serum uric acid levels, or hyperuricemia, have been shown to be associated with adverse clinical outcomes in patients with heart failure (HF). In HF, several mechanisms contribute to increased serum uric acid concentrations, including reduced uric acid clearance, the use of diuretic agents, and upregulation of xanthine oxidase expression within the myocardium, an enzyme involved in uric acid synthesis [21]. There are only a limited number of studies evaluating the prognostic role of uric acid in patients with AL amyloidosis. In a study published in 2008, Kumar et al. proposed that serum uric acid levels may represent a novel prognostic biomarker in AL amyloidosis [22]. However, hitherto no study has specifically investigated the prognostic significance of uric acid in patients with AL-CA.

In CA, amyloid deposition primarily causes diastolic dysfunction while LVEF is usually preserved until advanced disease stages. As reduced LVEF reflects more extensive myocardial involvement, it is unsurprising that previous studies have consistently identified low LVEF as an adverse prognostic marker [23]. However, from the early stages of the disease, LVGLS and MCF values begin to decrease [24]. In a study including both AL-CA and TTR-CA patients, MCF was identified as an independent predictor of mortality and demonstrated superior prognostic performance compared with LVEF [25,26]. In our study, although LVEF, GLS, and MCF were not identified as independent predictors in the multivariable analysis, they were significantly associated with overall survival in univariable analyses. Notably, GLS data were unavailable in 24 patients (25.0%), which, together with the relatively small sample size, may have reduced the statistical power to detect an independent association after multivariable adjustment. Therefore, the loss of statistical significance of GLS in the multivariable model should be interpreted with caution and should not necessarily be considered evidence of a lack of prognostic relevance. Consistent with the prognostic relevance of myocardial deformation parameters, Lei et al. demonstrated that speckle-tracking parameters, particularly GLS, were useful in predicting cardiac involvement and survival in AL amyloidosis, with GLS emerging as an independent predictor of survival [27]. Given the established association of GLS with the extent of myocardial involvement in cardiac amyloidosis, its independent prognostic value warrants further evaluation in larger, adequately powered cohorts with more complete echocardiographic data.

Beyond conventional and deformation-based imaging parameters, noninvasive pressure–volume (PV) loop analysis has emerged as a novel approach for assessing myocardial performance and may provide complementary prognostic information. More recently, Liang et al. evaluated CMR-derived PV loop parameters in patients with AL-CA. Reduced work efficiency was independently associated with all-cause mortality and provided incremental prognostic value when added to Mayo staging [28].

Finally, cardiac death in AL-CA is predominantly related to progressive heart failure and sudden cardiac death (SCD), with the latter potentially resulting from ventricular arrhythmias, bradyarrhythmias, or electromechanical dissociation [29,30]. The risk of SCD is particularly relevant in advanced cardiac involvement, although the benefit of implantable cardioverter–defibrillators for primary prevention remains uncertain [30]. As cause-specific mortality was not adjudicated in our study, future studies incorporating detailed rhythm monitoring and cause-specific endpoints may help clarify whether SBP, troponin levels, and NYHA functional class have differential prognostic implications for arrhythmic versus heart-failure-related mortality.

This study has several limitations, including its relatively small sample size and single-center design. The limited number of observed events constrained model complexity and may have reduced the precision and stability of the estimated associations. In addition, univariable screening followed by backward stepwise selection may have increased the risk of model instability and overfitting. Detailed treatment regimens and hematological or organ responses were not systematically available; therefore, potential confounding by treatment type, treatment eligibility, and treatment response cannot be excluded. Furthermore, the study period spanned 2014–2024, during which diagnostic and therapeutic practices for AL amyloidosis evolved, and residual confounding by calendar time remains possible. Missing data, particularly for advanced echocardiographic parameters, limited some analyses. Because a complete-case approach was used without imputation, missing data may also have introduced selection bias and reduced the statistical precision of the corresponding analyses. The proportional hazards assumption was not formally assessed for the Cox regression models; therefore, potential time-varying effects of the included covariates cannot be excluded. This should be considered when interpreting the reported hazard ratios. Although bootstrap resampling was used to estimate the confidence interval for the change in C-index, no optimism-corrected internal validation or external validation of the SBP-integrated approach was performed.

In conclusion, lower SBP, higher NYHA functional class, and marked troponin elevation were independently associated with mortality in this single-center cohort of patients with AL-CA. The addition of SBP to the Mayo 2012 staging system provided a modest incremental prognostic contribution. However, the SBP-integrated risk-stratification approach should be considered exploratory and requires prospective, multicenter external validation before it can be used for individual risk prediction or clinical decision-making.

## Figures and Tables

**Figure 1:**
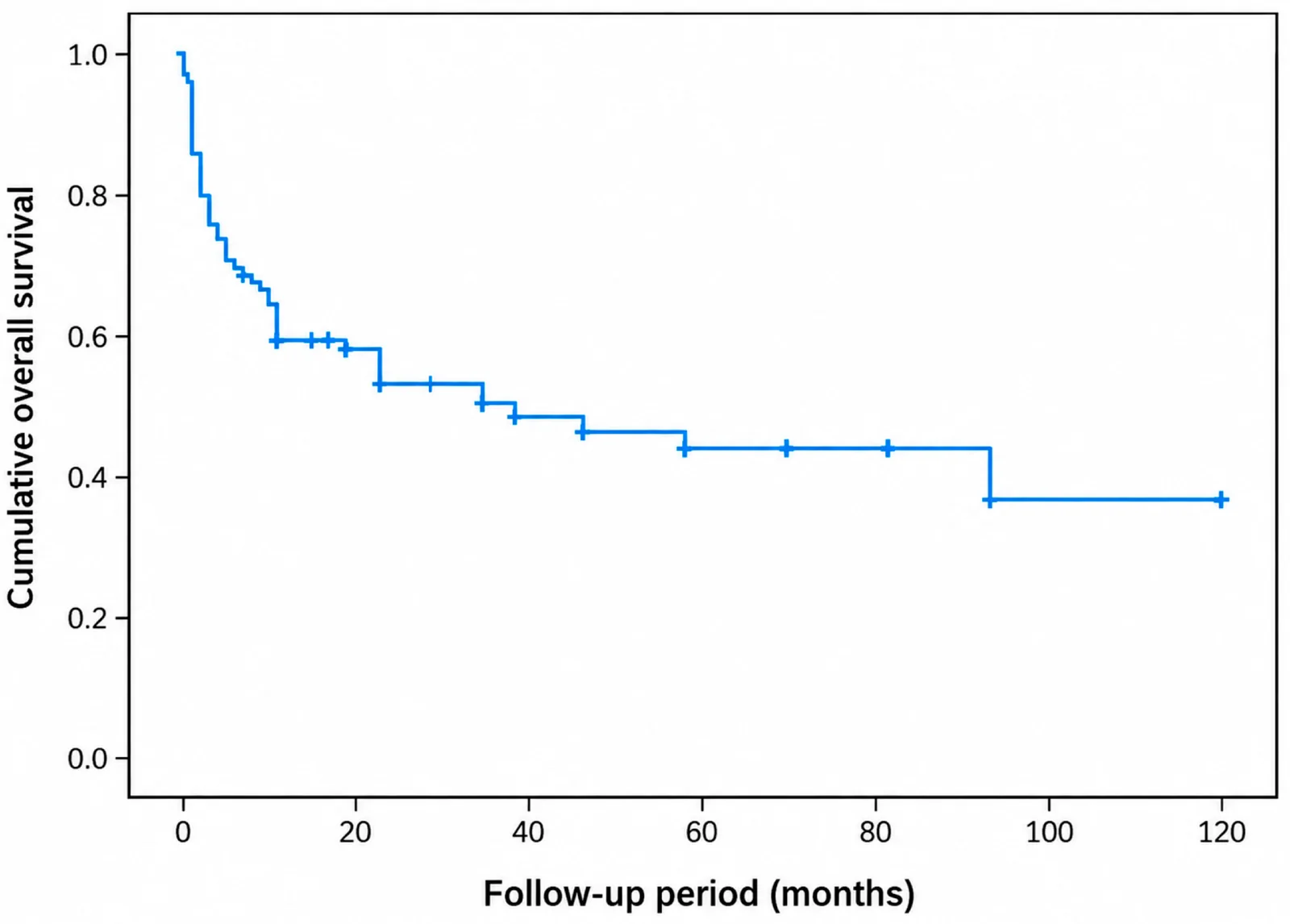
Kaplan–Meier curve showing cumulative overall survival in the AL-CA patients.

**Figure 2:**
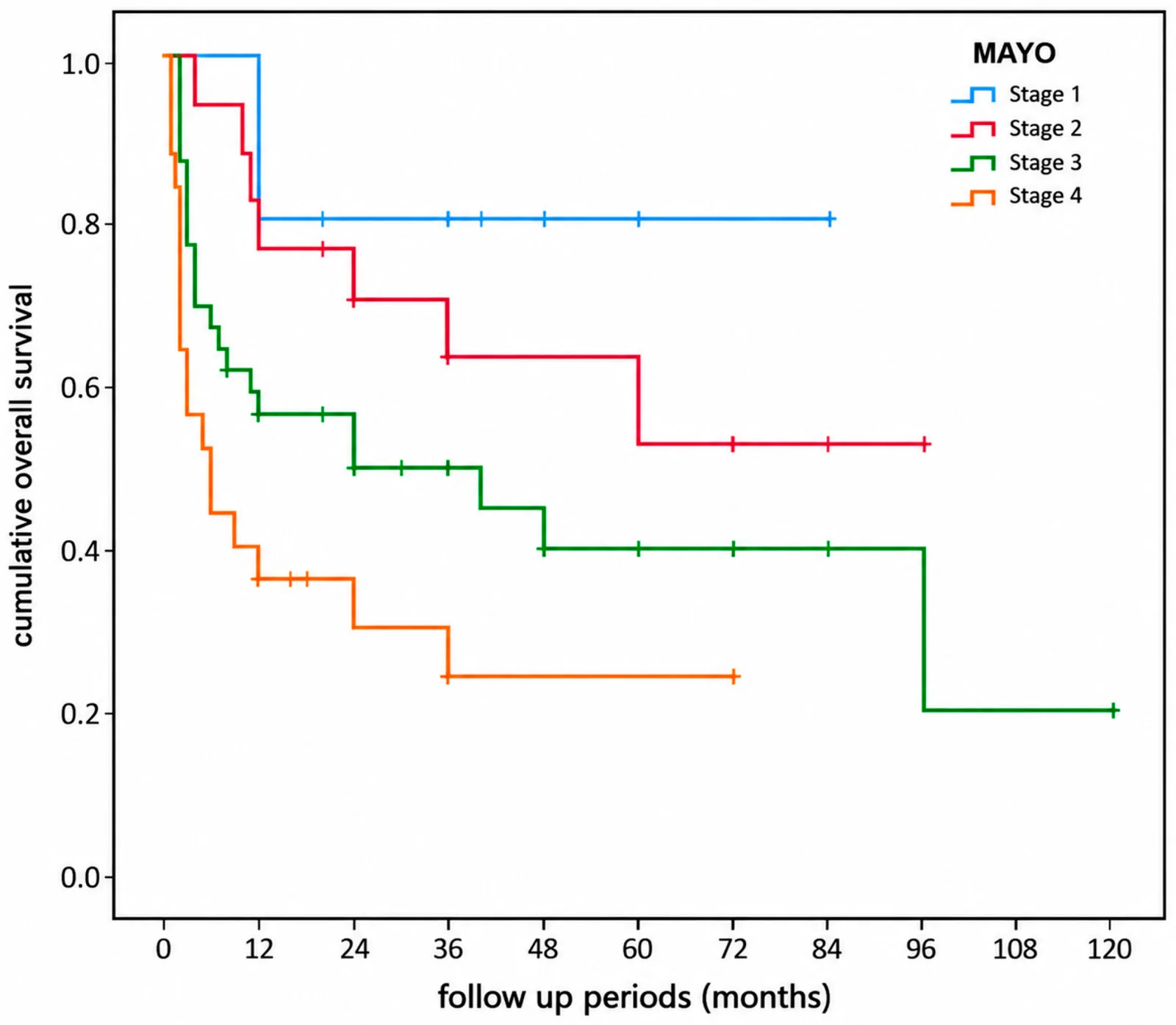
Kaplan–Meier curve showing overall survival according to Mayo stages.

**Figure 3:**
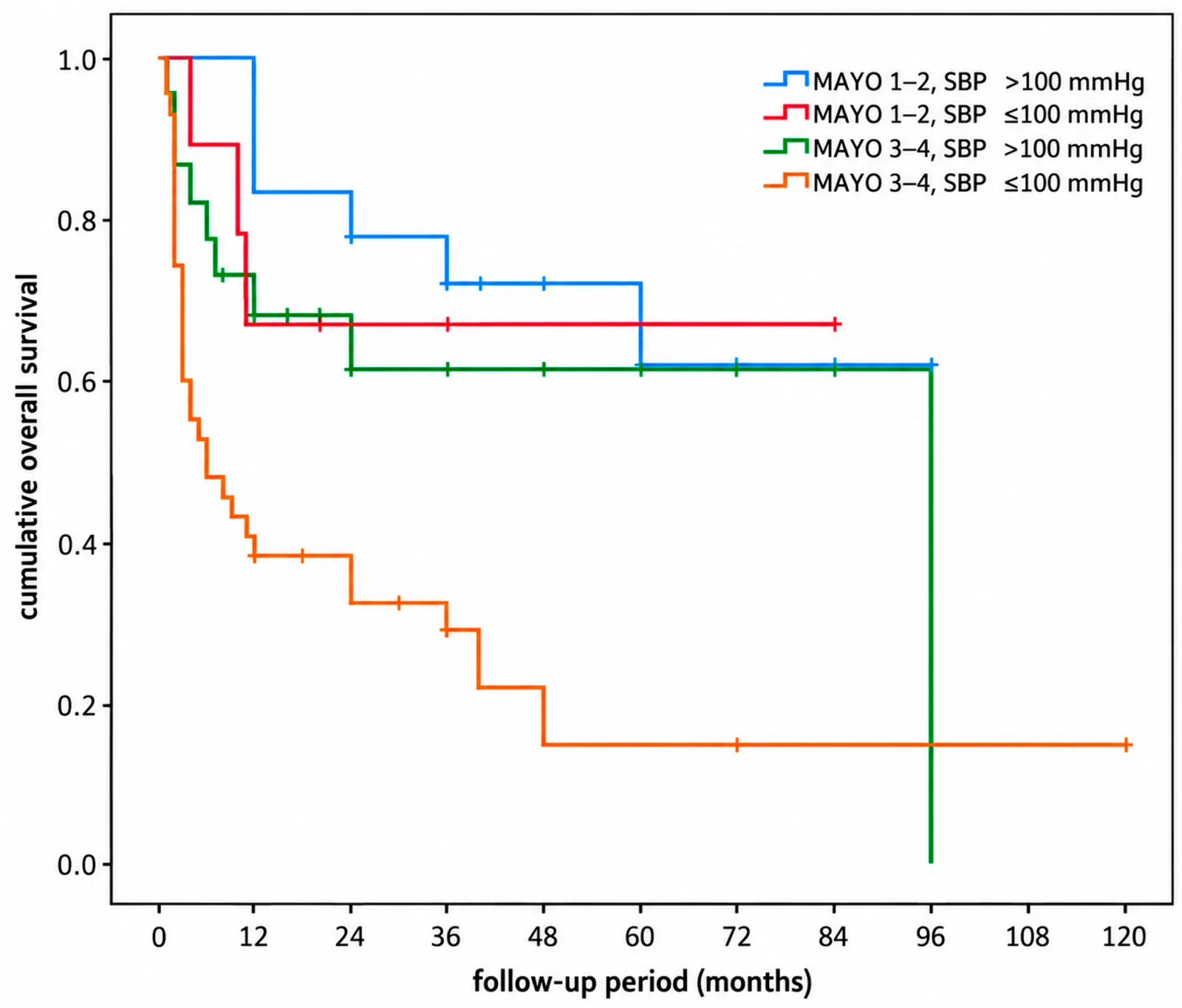
Kaplan–Meier survival curves according to the exploratory classification combining Mayo 2012 stage and systolic blood pressure.

**Table 1:** Descriptive statistics of demographic, hemodynamic, and laboratory parameters of the AL-CA patients.

	n = 96
**Demographic Data**	
Women, n (%)	38 (39.6)
Age at diagnosis	59.6 ± 11.2
SBP mmHg	100 (70–150)
DBP, mmHg	64 (40–90)
**Comorbidities, n (%)**	
Renal involvement	67 (69.8%)
Cerebrovascular disease	12 (12.5)
CTS	5 (5.2)
PNP	66 (68.7)
CAD	14 (14.6)
**Biochemical Parameters**	
hs-cTnT (0–14 ng/L)	72 (12–1795), n = 70
Troponin > 3× assay-specific URL, n (%)	62 (64.6)
BNP, (0–170 pg/mL)	2420.5 (378–2683), n = 4
NT-proBNP, (0–125 pg/mL)	2729.5 (77–35,000), n = 92
Uric acid, (2.4–5.7 mg/dL)	6.05 (2.1–19)
Creatinine (0.5–0.9 mg/dL)	1.09 (0.36–6)
**Electrocardiography, n (%)**	
AF	10 (10.4)
Presence of low-voltage criteria	42 (43.8)
**Plasma Cell Dyscrasia**	
Lambda light chain, n (%)	73 (76)
Multiple myeloma, n (%)	17 (17.7)
Serum free Kappa, (3.3–19.4 mg/L)	21.00 (5–17,500)
Serum free Lambda, (5.7–26.3) mg/L	128.00 (0.80–9600)
Kappa/Lambda ratio	0.14 (0.003–1750)
dFLC, mg/L	187.50 (4–17,490)
Bone marrow plasma cell ratio, %	12 (1–80)

Continuous variables are presented as mean ± standard deviation or median (minimum–maximum), as appropriate. Categorical variables are presented as n (%). The number of patients with available measurements is indicated for variables with missing data. **Abbreviations:** AF, atrial fibrillation; BNP, B-type natriuretic peptide; CAD, coronary artery disease; CTS, carpal tunnel syndrome; DBP, diastolic blood pressure dFLC, difference between involved and uninvolved free light chains; NT-proBNP, N-terminal pro-B-type natriuretic peptide; PNP, polyneuropathy; SBP, systolic blood pressure.

**Table 2:** Descriptive statistics of echocardiographic data.

	n = 96
LVEF, %	50.62 ± 10.17
IVS, mm	14.6 ± 2.40
LVPW, mm	13.7 ± 2.20
LA, mm	45.1 ± 6.20
Relative LV thickness	0.61 ± 0.15
LVEDD, mm	46.3 ± 5.7
LVESD, mm	31.7 ± 6.2
E/A	1.66 ± 1.12
E/e’	15.30 ± 6.65
Lateral e’, m/s	0.06 ± 0.02
Septal e’, m/s	0.05 ± 0.01
LVEF/LVGLS (n = 68)	4.41 ± 1.58
TAPSE, mm	16.63 ± 4.51
LVGLS, (-%) (n = 72)	−11.81 ± 5.64
SAB (n = 53)	4.03 ± 2.53
LV mass (g)	266.52 ± 70.27
Apical sparing, n (%) (n = 69)	61(88.4)
Pericardial effusion, n (%)	80 (83.3)

Data are presented as mean ± SD for continuous variables and as number (%) for categorical variables. The number of patients with available measurements is indicated for variables with missing data. **Abbreviations:** LVEF, left ventricular ejection fraction; IVS, interventricular septum; LVPW, left ventricular posterior wall; LA, left atrium; LVEDD, left ventricular end-diastolic diameter; LVESD, left ventricular end-systolic diameter; LVGLS, left ventricular global longitudinal strain; SAB, apical-to-basal strain ratio.

**Table 3:** Univariable Cox proportional hazards regression analysis results for all factors considered to potentially affect overall survival.

	Hazard Ratio	95% CI	*p*-Value
SBP, mmHg ^1^	1.42	1.19–1.70	**<0.001**
NYHA	2.73	1.72–4.33	**<0.001**
Troponin > 3× assay-specific URL	3.36	1.70–6.63	**<0.001**
NT-proBNP ^2^	1.01	0.98–1.04	0.265
NT-proBNP ≥ 1800 pg/mL	2.22	1.16–4.26	**0.017**
Uric acid	1.15	1.05–1.25	**0.002**
eGFR	0.99	0.98–1.00	0.141
Albumin	0.97	0.94–1.00	0.086
dFLC ^3^	1.04	1.00–1.09	**0.042**
Light chain type = Kappa	1.17	0.56–2.42	0.667

^1^ Effect of each 10 mmHg decrease in systolic blood pressure, ^2^ effect of each 1000-unit increase in NT-proBNP level, ^3^ effect of each 500-unit increase in dFLC level. **Abbreviations:** See Table 1.

**Table 4:** Univariable Cox proportional hazards regression analysis of echocardiographic and electrocardiographic parameters associated with overall survival.

	Hazard Ratio	95% CI	*p*-Value
LVEF	1.50	1.16–1.95	**0.002**
IVS	2.01	0.66–6.09	0.215
E/E’	1.01	0.97–1.05	0.520
Lateral E’	1.15	0.97–1.37	0.095
Septal E’	1.07	0.87–1.32	0.500
EF/LV strain	1.29	1.00–1.66	**0.045**
MCF	1.25	1.03–1.51	**0.020**
LVGLS	1.09	1.04–1.14	**<0.001**
Apical sparing	5.99	0.81–43.97	0.078
LV mass	0.99	0.99–1.00	0.390
SAB	1.04	0.87–1.23	0.651
AF	1.51	0.63–3.58	0.346
Low voltage	2.25	1.14–4.45	**0.019**

**Table 5:** Factors with the most significant predictive value for overall survival in the AL-CA group: results of multivariable backward stepwise Cox proportional hazards regression analysis (final model: n = 96; deaths = 51).

	Hazard Ratio	95% Confidence Interval		Wald	*p*-Value
		Lower Limit	Upper Limit		
**Model 1**					
SBP ^1^	1.28	1.06	1.53	7.18	**0.007**
NYHA	1.92	1.11	3.32	5.48	**0.019**
Troponin > 3 times elevation	2.51	1.17	5.42	5.58	**0.018**
Uric acid	1.01	0.91	1.12	0.12	0.727
LVEF ^2^	1.14	0.81	1.61	0.63	0.427
MCF	1.01	0.96	1.06	0.17	0.677
**Model 2**					
SBP ^1^	1.29	1.08	1.53	8.24	**0.004**
NYHA	1.95	1.14	3.33	6.08	**0.014**
Troponin > 3 times elevation	2.53	1.18	5.44	5.70	**0.017**
LVEF ^2^	1.14	0.81	1.61	0.64	0.422
MCF	1.00	0.96	1.05	0.12	0.722
**Model 3**					
SBP ^1^	1.28	1.08	1.52	8.20	**0.004**
NYHA	1.92	1.14	3.25	6.00	**0.014**
Troponin > 3 times elevation	2.49	1.16	5.31	5.55	**0.018**
LVEF ^2^	1.11	0.82	1.51	0.51	0.472
**Model 4**					
SBP ^1^	1.30	1.10	1.54	10.05	**0.002**
NYHA	1.92	1.14	3.24	6.05	**0.014**
Troponin > 3 times elevation	2.67	1.29	5.55	6.98	**0.008**

^1^ Effect of every 10 mmHg decrease in systolic blood pressure. ^2^ Effect of every 10-unit decrease in EF. **Abbreviations:** See Table 1 and Table 2.

## Data Availability

The data supporting the findings of this study are not publicly available due to patient privacy and ethical restrictions. De-identified data may be made available by the corresponding author upon reasonable request, subject to institutional and ethical approval.
